# Less Expression of Prohibitin Is Associated with Increased Paired Box 2 (PAX2) in Renal Interstitial Fibrosis Rats

**DOI:** 10.3390/ijms13089808

**Published:** 2012-08-06

**Authors:** Tian-Biao Zhou, Zhi-Yu Zeng, Yuan-Han Qin, Yan-Jun Zhao

**Affiliations:** 1 Department of Pediatrics, The First Affiliated Hospital of Guangxi Medical University, Nanning 530021, China; E-Mails: a126tianbiao@126.com (T.-B.Z.); yanjunzhao08@126.com (Y.-J.Z.); 2 Department of Cardiology/Geriatrics, The First Affiliated Hospital of Guangxi Medical University, Nanning 530021, China; E-Mail: zhiyuzeng@yeah.net

**Keywords:** prohibitin, PAX2, TGF-β1, renal interstitial fibrosis, cell apoptosis

## Abstract

Prohibitin (PHB) and paired box 2 (PAX2) are associated with the development of renal interstitial fibrosis (RIF). This study was performed to investigate whether or not the PHB could regulate the PAX2 gene expression in unilateral ureteral obstruction (UUO) in rats. Eighty Wistar male rats were randomly divided into two groups: sham operation group (SHO) and model group subjected to unilateral ureteral obstruction (GU), *n* = 40, respectively. The model was established by left ureteral ligation. Renal tissues were collected at 14-day and 28-day after surgery. RIF index, protein expression of PHB, PAX2, transforming growth factor-βl (TGF-β1), α-smooth muscle actin (α-SMA), collagen-IV (Col-IV), fibronectin (FN) or cleaved Caspase-3, and cell apoptosis index in renal interstitium, and mRNA expressions of PHB, PAX2 and TGF-β1 in renal tissue were detected. When compared with those in SHO group, expression of PHB (mRNA and protein) was significantly reduced, and expressions of PAX2 and TGF-β1 (protein and mRNA) were markedly increased in the GU group (each *p* < 0.01). Protein expressions of α-SMA, Col-IV, FN and cleaved Caspase-3, and RIF index or cell apoptosis index in the GU group were markedly increased when compared with those in the SHO group (each *p* < 0.01). The protein expression of PHB was negatively correlated with protein expression of PAX2, TGF-β1, α-SMA, Col-IV, FN or cleaved Caspase-3, and RIF index or cell apoptosis index (all *p* < 0.01). In conclusion, less expression of PHB is associated with increased PAX2 gene expression and RIF index in UUO rats, suggesting that increasing the PHB expression is a potential therapeutic target for prevention of RIF.

## 1. Introduction

Renal interstitial fibrosis (RIF) is the major histopathological change seen in a variety of renal disorders and is closely related to renal dysfunction [1]. Tubule-interstitial changes, including tubular degeneration and interstitial cell infiltration, are a hallmark of common progressive chronic diseases that lead to renal failure [2]. The elevation of transforming growth factor-β1 (TGF-β1), α-smooth muscle actin (α-SMA) and extracellular matrix (ECM) in renal interstitium is the most important feature of RIF. However, the exact mechanism is complicated and is not well elucidated at the moment.

Prohibitin (PHB, also known as PHB1), a member of the Band-7 family of proteins, is highly conserved evolutionarily, widely expressed, and present in different cellular compartments [3]. It plays a pivotal role in the processes of cell differentiation and apoptosis [4]. The over-expression of PHB could protect the mitochondria from oxidative stress-induced injury [5]. When the function of mitochondria is confused, more reactive oxygen species (ROS) will be released from the mitochondria. ROS from mitochondria is a strong agent to induce the expression of TGF-β1 and promotes TGF-β1-dependent myofibroblast differentiation [6]. Over-expression of PHB could suppress renal interstitial fibroblasts proliferation and halt the progression of RIF [7]. So, PHB might take part in the development and progression of RIF.

Paired box gene 2, PAX2, encodes for a transcription factor that is up-regulated during nephrogenesis and becomes silenced in mature epithelium of the glomeruli, the proximal tubule, and distal tubule [8,9]. PAX2 is essential for development of the urogenital system, embryo, inner ear, neural tube, optic vesicle, optic cup and optic tract [10–12]. PAX2 has been implicated as an oncogene in carcinomas of the kidney, prostate, breast, ovary and so on [13]. Interestingly, ROS can induce the PAX2 expression [14,15] and the over-expression of PAX2 is found in the renal tissue suffering from RIF [16]. PAX2 is associated with the development of RIF.

From the above (PHB or PAX2 was associated with ROS, furthermore, PHB or PAX2 was associated with RIF), we drew a hypothesis that there was an association between PHB and PAX2 in the progression of RIF. This investigation was conducted to explore whether or not PHB was associated with the gene expression of PAX2 in UUO rats.

## 2. Results

### 2.1. Renal Morphology

**Figure 1:**
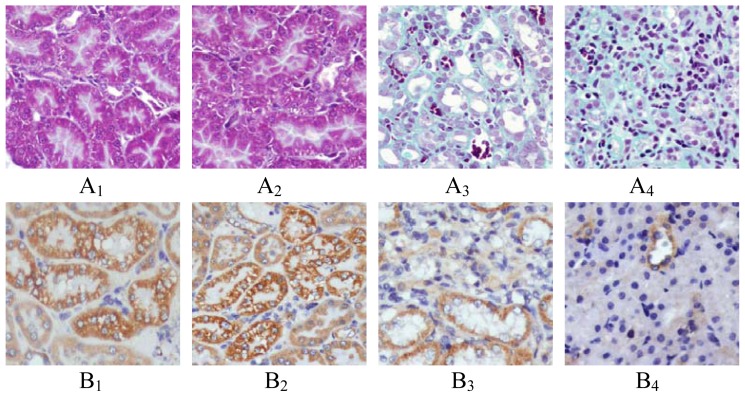

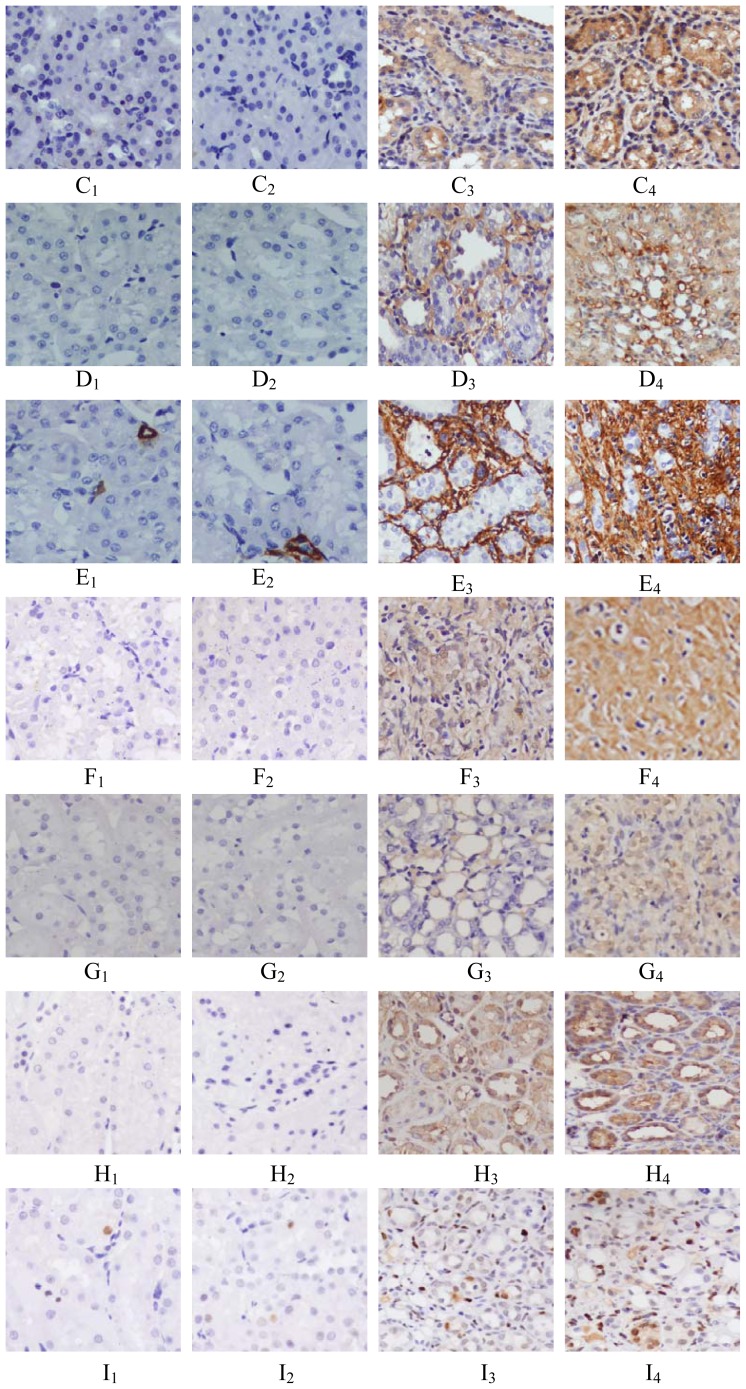
Tissue parameters in two groups. Masson staining for SHO group (A_1_: 14-day; A_2_: 28-day) and GU group (A_3_: 14-day; A_4_: 28-day). Renal morphology was normal in sham group (A_1_ and A_2_). Renal tubular structure was severely, collapsed lumen, diffusive infiltration of fibroblast in renal interstitium, and collagen formation in the majority of extracellular matrix in GU group (blue = collagen) (A_3_ and A_4_), especially in A_4_. Representative samples of immunohistochemical staining for PHB (SHO: B_1_: 14-day, B_2_: 28-day; GU: B_3_: 14-day, B_4_: 28-day); PAX2 (SHO: C_1_: 14-day, C_2_: 28-day; GU: C_3_: 14-day, C_4_: 28-day); TGF-β1 (SHO: D_1_: 14-day, D_2_: 28-day; GU: D_3_: 14-day, D_4_: 28-day); α-SMA (SHO: E_1_: 14-day, E_2_: 28-day; GU: E_3_: 14-day, E_4_: 28-day); Col-IV (SHO: F_1_: 14-day, F_2_: 28-day; GU: F_3_: 14-day, F_4_: 28-day); FN (SHO: G_1_: 14-day, G_2_: 28-day; GU: G_3_: 14-day, G_4_: 28-day), cleaved Caspase-3 (SHO: H_1_: 14-day, H_2_: 28-day; GU: H_3_: 14-day, H_4_: 28-day) and cell apoptosis (SHO: I_1_: 14-day, I_2_: 28-day; GU: I_3_: 14-day, I_4_: 28-day) were observed in two groups. The staining for PHB in GU group (B_3_ and B_4_) was markedly reduced when compared with that in SHO (B_1_ and B_2_), especially in 28-day. However, the PAX2 staining in GU group (C_3_ and C_4_) was much more remarkable when compared with that in SHO group (C_1_ and C_2_), especially in C_4_. The staining for PHB and PAX2 were mainly located in RTEC. Positive stainings (in brown) for TGF-βl, α-SMA, Col-IV and FN were stronger in the area of extracellular matrix in GU group than those in SHO group, especially in 28-day of GU group. The staining for cleaved Caspase-3 in GU group (H_3_ and H_4_) was much remarkably when compared with that in SHO group (H_1_ and H_2_), especially in H_4_. Cleaved Caspase-3 was also mainly located in the RTEC and the apoptotic cell in our observation was mainly derived from RTEC. PHB: prohibitin; PAX2: paired box 2; TGF-β1: transforming growth factor-βl; α-SMA: α-smooth muscle actin; Col-IV: collagen-IV; FN: fibronectin; RTEC: renal tubular epithelial cells; SHO: sham operation group; GU: model group subjected to unilateral ureteral obstruction. Magnification × 400.

**Figure 2:**
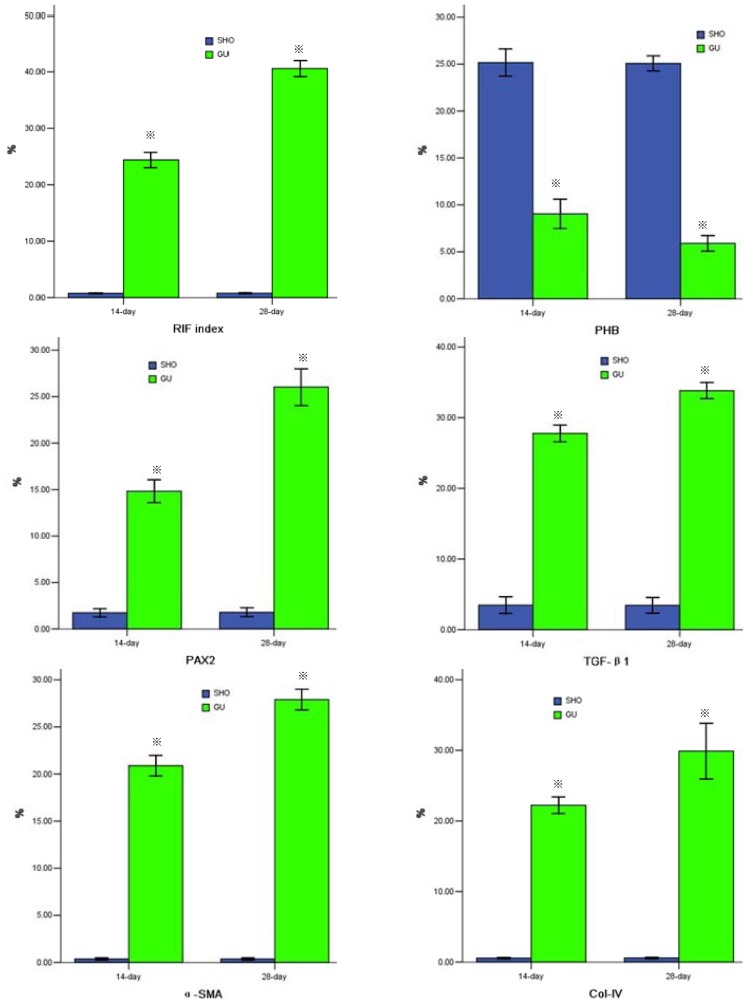

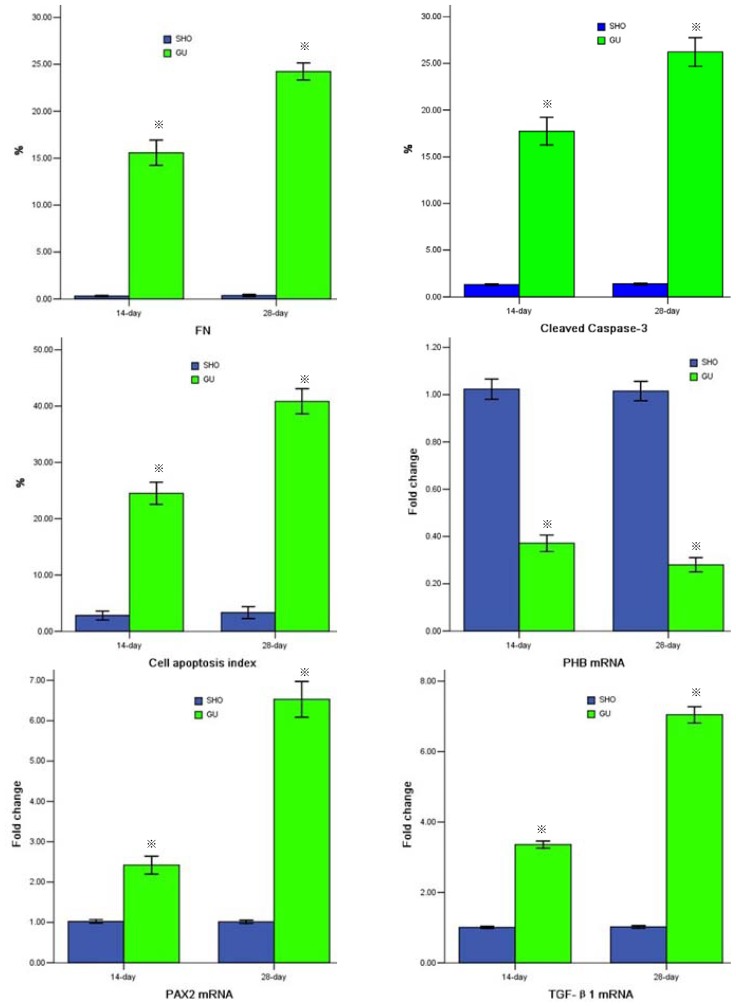
Statistical parameters in two groups. ※ *p* < 0.01 compared with SHO. SHO: sham operation group; GU: model group subjected to unilateral ureteral obstruction; RIF: renal interstitial fibrosis; PHB: prohibitin; PAX2: paired box 2; TGF-β1: transforming growth factor-βl; α-SMA: α-smooth muscle actin; Col-IV: collagen-IV; FN: fibronectin.

More collagen deposition, fibroblast proliferation and diffused lymphoeytein filtration in the renal interstitium of GU group were observed when compared with those in SHO group (Figure 1). The index of RIF in GU was notably elevated when compared with that in SHO (*p* < 0.01; Figure 2).

### 2.2. Protein Expression of PHB, PAX2, TGF-βl, α-SMA, Col-IV, FN or Cleaved Caspase-3

When compared with SHO, in GU group the protein expression of PHB in renal interstitium was significantly weakened (*p* < 0.01, Figures 1 and 2) and the protein expressions of PAX2, TGF-βl, α-SMA, Col-IV, FN and cleaved Caspase-3 in renal interstitium were significantly increased (all *p* < 0.01, Figures 1 and 2). PHB, PAX2 and cleaved Caspase-3 were mainly located in the renal tubular epithelial cells (RTEC) in our observation (Figure 1).

### 2.3. Cell Apoptosis

The staining for cell apoptosis was much more significant in renal interstitium in GU group than that in SHO group (Figure 1), and the apoptosis index was significantly increased in GU group when compared with that in SHO (*p* < 0.01, Figure 2). Interestingly, the apoptotic cell in our observation was mainly derived from RTEC (Figure 1).

### 2.4. mRNA Expression of PHB, PAX2 or TGF-βl

**Figure 3:**
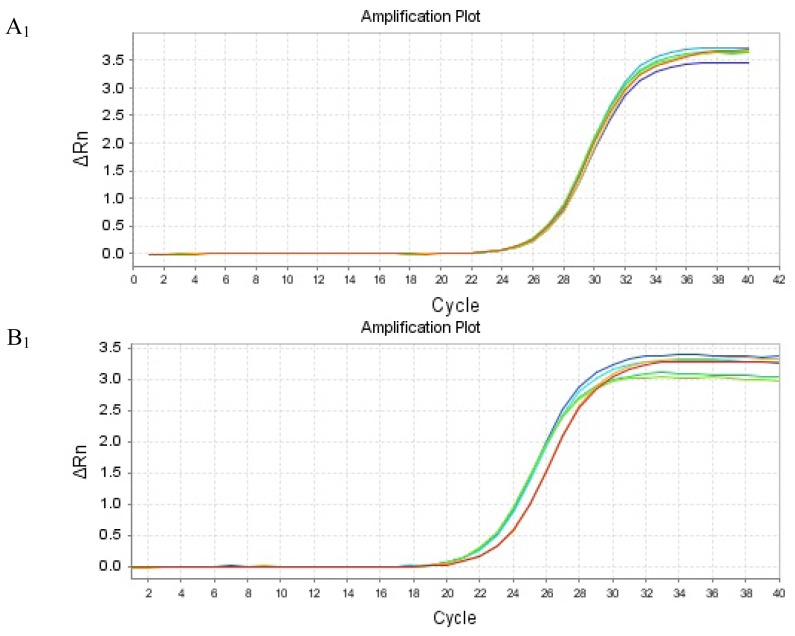

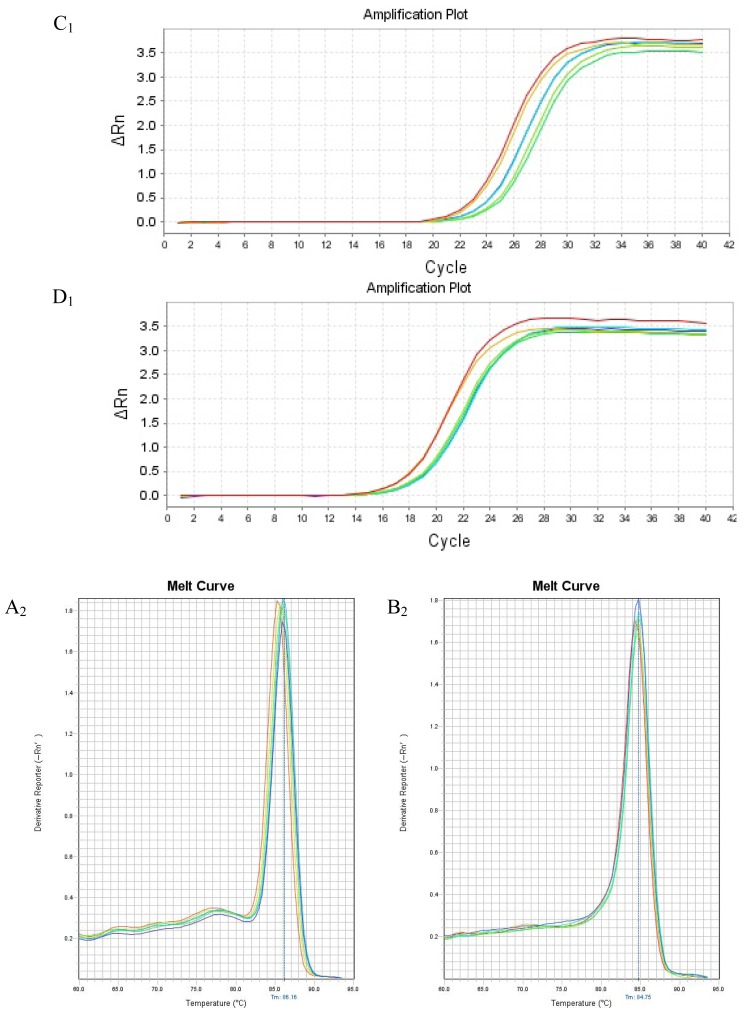

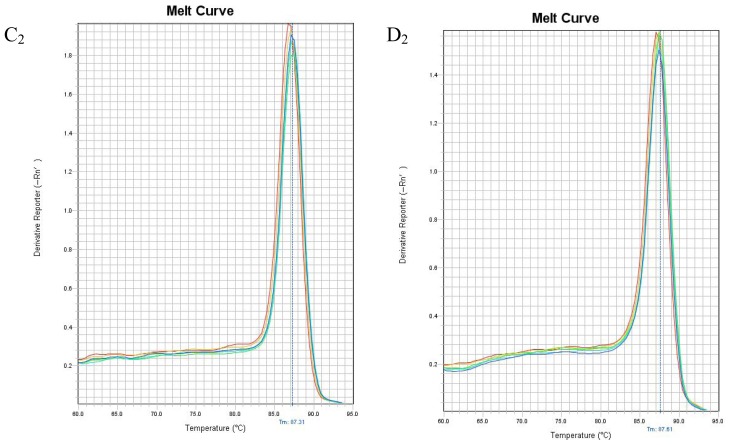
Amplification curve and melting curve for PHB, PAX2, TGF-βl and β-actin. A_1_: amplification curve for PHB; A_2_: melting curve for PHB; B_1_: amplification curve for PAX2; B_2_: melting curve for PAX2; C_1_: amplification curve for TGF-βl; C_2_: melting curve for TGF-βl; D_1_: amplification curve for β-actin; D_2_: melting curve for β-actin.

Renal tissue of GU group showed consistently lower PHB mRNA expression and higher PAX2 or TGF-βl mRNA expression, when compared to those in SHO respectively (all *p* < 0.01; Figure 2). The amplification curve and melting curve for PHB, PAX2, TGF-βl and β-actin were shown in Figure 3.

### 2.5. Correlation Analysis

There was a negatively correlation between PHB protein and index of RIF, protein expression of PAX2, TGF-βl, α-SMA, Col-IV, FN or cleaved Caspase-3, or cell apoptosis index (*r* = −0.825, −0.798, −0.817, −0.786, −0.948, −0.953, −0.863, −0.886; each *p* < 0.01). PAX2 protein level was positively correlated with RIF index, TGF-βl, α-SMA, Col-IV, FN or cleaved Caspase-3, or cell apoptosis index (*r* = 0.732, 0.833, 0.864, 0.757, 0.837, 0.902, 0.886; each *p* < 0.01).

## 3. Discussion

RTEC, an important cell taking part in the process of RIF, suffers from ischemic injury [17,18] which can increase the production of ROS, and undergoes epithelial-mesenchymal transition (EMT) in RIF induced by UUO [19,20]. Over-expression and deposit of ECM, such as Col-IV and FN, are the important characteristics of RIF. RTEC suffering from EMT plays a crucial role in the progress of RIF [21,22]. α-SMA, as a specific marker for EMT, takes part in the development and progression of RIF [21,23]. Of all the cytokines and growth factors involved, TGF-β1 plays the most important role when compared with others, and the increased expression of TGF-β1 is closely correlated with the development of RIF [24,25]. TGF-β1 is known to be one of the major mediators and leads to RIF by inducing the production of α-SMA and ECM (Col-IV and FN) in renal interstitium [26–28]. So, TGF-β1, α-SMA, Col-IV and FN are the important indicators to evaluate the grade of RIF lesion and the progression of RIF. Cleaved Caspase-3 is a pivotal effector of the apoptosis machinery [29] and cleaved Caspase-3 activity was associated with cell apoptosis [30,31]. Cell apoptosis is most important for the development of RIF [32–34]. In this investigation, those indicators were evaluated.

In this study, we found that index of RIF, mRNA and protein expression of PAX2 or TGF-βl, protein expression of α-SMA, Col-IV, FN or cleaved Caspase-3, or cell apoptosis index were markedly increased in GU group when compared with those in SHO group, especially in 28-day. We also found the impaired RTEC was the main contributor for RIF in UUO model. A conclusion could confidently be drawn that the RIF model induced by UUO in our study was successful. However, the pathological mechanism of RIF was not elucidated.

PHB is a highly conserved, ubiquitously expressed protein that participates in diverse processes including mitochondrial chaperone, growth and apoptosis [35] and is regarded as an apoptosis-regulating protein [36]. The PHB might have played a protective role against injury in cells or tissue in some studies. Ko *et al.* [35] found that hepatocyte-specific PHB deficiency resulted in marked liver injury, oxidative stress, and fibrosis with development of hepatocellular carcinoma, suggesting that PHB was a tumor suppressor in hepatocytes. Liu *et al.* [5] conducted a study in cardiomyocytes and their data indicated that PHB could protect the cardiomyocytes from oxidative stress-induced damage, and that increasing PHB content in mitochondria constituted a new therapeutic target for myocardium injury. Muraguchi *et al.* [37] performed an investigation in H9C2 cardiomyocytes and found that PHB might function as a survival factor against hypoxia-induced cell death. The results from the above mentioned studies drew a consistent conclusion that PHB could protect the cells or tissue from ROS-induced injury.

PHB might be expressed in renal tissue. There were also some observations found that PHB might be observed in renal tissue and that PHB might play a protective role in kidney against renal diseases. Guo *et al.* [7] observed that PHB protein was positively expressed in normal renal tissues, strongly down-regulated in renal biopsy specimens from patients, and negatively correlated with the expression of a-SMA and with the degrees of tubulointerstitial lesions. They also conducted a study in rat kidney fibroblasts cell line and found over-expression of PHB suppresses renal interstitial fibroblasts proliferation and cell phenotypic change induced by TGF-βl. Wu *et al.* [38] performed a study in rats with renal tubular atrophy and interstitial fibrosis induced by aristolochic acid and found that the expression of PHB protein was down-regulated in renal tissue of rats. Quan *et al.* [39] observed that the expression of Prohibitin-2 (homologue of PHB1 [40]) was down-regulated in RETC stimulated by elevated uric acid, which may promote trans-differentiation of renal tubular epithelial cells. Those reports consistently agreed that PHB was a protective factor, which is similar to our results. In this study, we found that PHB was mainly located in RTEC and was correlated negatively with protein expression of TGF-βl, α-SMA, Col-IV, FN or cleaved Caspase-3, index of RIF or cell apoptosis index. The PHB expression in normal control group was more notable when compared with that in GU group. In conclusion, PHB can suppress the development of RIF by alleviating the expression of TGF-βl (mRNA and protein), the protein expression of α-SMA, Col-IV, FN or cleaved Caspase-3, and can weaken the indexes of RIF and cell apoptosis.

PAX2, a nuclear transcription factor, was recently observed in renal interstitium and takes part in the development and progression of RIF. Normal expression of PAX2 is necessary for the regular development of renal tissue and kidney cells [41–43]. Some reports found that the expression of PAX2 was closely associated with some kidney diseases. Li *et al.* [44] conducted a study in rats with obstructive nephropathy and found that PAX2 was re-expressed in the renal tubules, which might participate in the pathogenesis of renal tubular damage and RIF. The result from Li *et al.* was similar to the result obtained in our investigation. Benetti *et al.* [45] performed a study of a 13-year-old boy suffering from familial juvenile hyperuricemic nephropathy and a renal biopsy showed that PAX2-positive immunostaining was notably seen at the corticomedullary junction and most of glomeruli featuring an enlargement of Bowman space (glomerular cysts), with mild interstitial fibrosis, inflammatory infiltrate, and focal tubular atrophy at the cortical level. Mure *et al.* [46] in a study of 26 fetal lambs performed surgical UUO and found that PAX2 protein was highly expressed in the nephrogenic zone, decreasing progressively to being markedly decreased in control lamb kidneys. Murer *et al.* [16] investigated 17 biopsies of juvenile nephronophthisis and observed that the failure of PAX2 repression involved in interstitial fibrosis and cysts formation. Huang *et al.* [47] explored the potential role of PAX2 in EMT that was induced in the remnant kidney of rats following 5/6 nephrectomy, and also examined PAX2 in cultured renal tubular epithelial cells, and their interesting experiments suggest that the re-expression of PAX2 in the tubular epithelial cells played an important role in the promotion of EMT, and there might a therapeutic value in silencing PAX2 to prevent or reverse renal fibrosis. Furthermore, ROS can induce PAX2 expression [14,15] and over-expression of PAX2 is found in the renal tissue suffering from RIF [16]. PAX2 is associated with the development of RIF. In conclusion, PAX2 might be involved in the RIF.

In our study, we found that PHB was correlated negatively with PAX2. The PHB expression (mRNA or protein) in SHO group was much more remarkable than that in the GU group, and the PAX2 gene expression (mRNA or protein) in SHO group was markedly attenuated when compared with that in GU group. We speculated on the following mechanism: The PHB expression in GU group was weakened, which induced the generation of ROS [5]. The increased ROS might up-regulate the gene expression of PAX2 [14,15], which regulated the expression of TGF-βl [48,49]. The disorder of TGF-βl might induce the expression of α-SMA, Col-IV and FN, and the increased TGF-βl could up-regulate the expression of cleaved Caspase-3 [50–52]. The over-expression of cleaved Caspase-3 was associated with cell apoptosis. Hence, the over-accumulation of ECM was observed and indexes of RIF and cell apoptosis were increased.

There was a rare report investigating whether or not PHB could affect the gene expression of PAX2. We found that less expression of PHB was associated with the increased gene expression of PAX2. The elevated expression of PAX2 took part in the development of RIF. We drew a hypothesis that there was a signal pathway between PHB and PAX2, and PHB could down-regulate the gene expression of PAX2 and could play a beneficial effect in the process of RIF. But, the detailed mechanism was not clear and the signal pathway in our hypothesis should be confirmed *in vitro* in the future.

Interestingly, as a nuclear transcription factor, the immunohistochemical staining points of PAX2 should gather in the nucleus, but the staining points of PAX2 could be found in cytoplasm in our study. Questions which need to be address in the future include: Whether the distribution of PAX2 was changed in the progression of renal interstitial fibrosis and how the PAX2 in the cytoplasm had a biological effect.

## 4. Materials and Methods

### 4.1. Animal Model

The Animal Care and Use Committee of Guangxi Medicial University approved all protocols. Eighty Wistar male rats (6-week-old) were purchased from the Experimental Animal center of Guangxi Medicial University, Nanning, China. The rats were randomly divided into two groups: sham operation group (SHO) and model group subjected to unilateral ureteral obstruction (GU); *n* = 40, respectively. The ureter was ligated at approximately 1 cm below the renal hilum with 3-0 silk suture. The abdominal wound was closed, and rats returned to the cages. Control rats underwent abdominal incision and approximation with no ligation of the ureter [53,54]. Twenty rats of the two groups were killed on 14-day and 28-day after surgery respectively and their renal tissue were collected for histological and molecular biology determination.

### 4.2. Renal Morphology

Sections were prepared on a microtome and stained with Masson’s trichrome staining and the RIF index was calculated using semi-quantitative evaluation as our previous description [55].

### 4.3. Immunohistochemical Analysis of the Protein Expressions of PHB, TGF-β1, α-SMA, Collagen-IV (Col-IV), Fibronectin (FN) and Cleaved Caspase-3

Renal tissue fixed with 4% buffered paraformaldehyde was embedded in paraffin, and 4 μm thick sections were stained. The positive area was measured quantitatively using a computer-aided manipulator (DMR+Q550, Leica Co., Wetzlar, Germany). For immunohistochemical analysis of PHB, TGF-β1, Col- IV, FN and cleaved Caspase-3, the sections were deparaffinized, washed with PBS, and treated with 3% H_2_O_2_ in methanol for 10 min. All sections were then incubated with anti-PHB antibody (1:300) (Neomarker lab, Co., Fremont, NE, USA), anti-PAX2 antibody (1:100) (ABSUN, Co., Missouri, MO, USA), anti-TGF-β1 antibody (1:100) (Zhongshan, Co., Beijing, China), anti-α-SMA antibody (ready-to-use kit) (Shanghai Changdao, Co., Inc., China), anti-Col-IV antibody (ready-to-use kit) (Bo Shide, Co., Wuhan, China), anti-FN antibody (1:50) (Zhongshan, Co., Beijing, China) and anti-cleaved Caspase-3 antibody (1:200) (Thermo Fisher Scientific, Co., Runcorn, UK), respectively. After incubation with second antibody immunoglobulin (Shanghai Changdao, Co., Shanghai, China), the sections were stained with diaminobenizidine (Maixin Bio, Co., Fuzhou, China). The staining of PHB, PAX2, TGF-βl, Col-IV, FN or cleaved Caspase-3 in renal tissue was measured. During evaluation of the interstitial areas, fields containing glomerular parts were ignored. All the evaluations were performed by two of the authors blinded to the experimental code.

### 4.4. Apoptosis Assay

Cell apoptosis was examined by the TdT mediated dUTP nick end labelling (TUNEL) assay (Roche Inc., Basel, Switzerland) as described previously [56,57]. Six slides from each kidney were evaluated for percentage of apoptotic cells by using the TUNEL assay. Then 10 watch fields, which didn’t include the glomerular parts, were chosen at random under microscope on each section. Brown staining of cell nuclei was considered apoptotic cells. Positive brown cells and total cells were counted. The formula for apoptosis index as the indicator of apoptosis was as follow [58–60]: cell apoptosis index = positive cells/total cells × 100%. The scores obtained by two investigators were averaged.

### 4.5. Real Time Reverse Transcription Polymerase Chain Reaction to Detect PHB, PAX2 and TGF-β1 mRNA Expressions in Renal Tissue

Total RNA was extracted from renal tissue using TRIzol (Beijing Tiangen, Co., China), and the operation procedure and method of calculation were the same as our previous description [55]. The primers for PHB, PAX2, TGF-βl and internal control β-actin were as follows: F 5′-TGGCGTTAGCGGTTACAGGAG-3′ and R 5′-GAGGATGCGTAGTGTGATGTTGAC-3′ for PHB; F 5′-AAGCGACAGAACCCGACTATGT-3′ and R 5′-ACTCCTGTCCCTGCCCCAT-3′ for PAX2; F 5′-TGAGCACTGAA GCGAAAGCC-3′ and R 5′-GATTCAAGTCAACTGTGGAGCAAC-3′ for TGF-βl; F 5′-GCCCCTGAGGAGCACCCTGT-3′ and R 5′-ACGCTCGGTCAGGATCTTCA-3′ for β-actin. Real time reverse transcription polymerase chain reaction was performed on Applied Biosystems 7500 Fast Real-Time PCR System using SYBR Green as fluorescent dye [61,62]. The mRNA expression of PHB, PAX2, or TGF-βl was calculated as our previous description [63–65].

### 4.6. Statistical Analysis

The data are shown as mean ± standard deviation. Independent-Samples *T* Test was performed to determine the differences between SHO group and GU group, and the Pearson’s correlation coefficient was used to determine the relationships between the indicators for detection. A value of *p* < 0.05 was considered as significant [66]. Statistical analysis was performed using the statistical package for social studies SPSS version 13.0 (SPSS, Chicago, IL, USA, 2004).

## 5. Conclusions

In conclusion, PHB appeared to play a protective role against RIF in UUO rats in our study, and PHB was associated with the gene expression of PAX2 and could alleviate the accumulation of ECM in renal interstitium; although the detailed mechanisms were not fully understood. Therefore, how to up-regulate the expression of PHB and down-regulate the PAX2 expression is very important for prevention of RIF, and PHB might be a potential therapeutic target for prevention of cell injury. This observation might offer some new insights to prevent the progression of RIF. However, cells culture in RTEC and so on, and inhibition of signaling pathway for PHB and PAX2 all need to be further investigated to explore the detailed mechanisms.
